# Realization of ongoing evolutionary adaptation in the field

**DOI:** 10.1093/evlett/qrag013

**Published:** 2026-04-11

**Authors:** Ruth G Shaw, Charles J Geyer, Mason W Kulbaba, Seema N Sheth, Vincent M Eckhart, Rachel E Pain

**Affiliations:** Department of Ecology, Evolution and Behavior, University of Minnesota-TC, St. Paul, MN, United States; School of Statistics, University of Minnesota-TC, Minneapolis, MN, United States; Department of Ecology, Evolution and Behavior, University of Minnesota-TC, St. Paul, MN, United States; Natural Science and Mathematics, St. Mary’s University, Calgary, Canada; Department of Ecology, Evolution and Behavior, University of Minnesota-TC, St. Paul, MN, United States; Department of Plant and Microbial Biology, North Carolina State University, Raleigh, NC United States; Department of Biology, Grinnell College, Grinnell, IA United States; Department of Ecology, Evolution and Behavior, University of Minnesota-TC, St. Paul, MN, United States; Minnesota Department of Natural Resources, St. Paul, MN United States

**Keywords:** additive genetic variance, aster models, *Chamaecrista fasciculata*, fundamental theorem of natural selection, genotype–environment interaction

## Abstract

The rapid pace of environmental change has prompted pressing concerns about the persistence of wild populations. For plants, because they move from one place to another only passively between generations, their persistence is especially likely to depend on their capacity for ongoing adaptive evolution. There are numerous examples of rapid adaptation in the recent past, but evidence about rates of adaptation in the wild is limited. Previously, to assess the capacity for genetic adaptation of three wild plant populations growing in their source locations, we have estimated their additive genetic variance for fitness in three successive years. Here, we present the actual difference between successive generations in their average absolute fitness. We partition this change into components resulting from genetic change and due to environmental difference, as well as a residual component. In each of six cases of intergenerational change, we have detected evolutionary adaptation as genetic increase in average fitness during the first generation, while also finding generally greater effects of differences in environment between years. Nevertheless, we show that when environmental change reduces a population’s average fitness, these adaptive genetic responses often substantively ameliorate its deleterious impact.

## Teaser

Genetic variation in fitness is understood as the basis of evolutionary adaptation, but the intergenerational genetic change in mean fitness is poorly known. Here, by directly evaluating fitness of pedigreed sets of individuals growing in the field, we estimated the adaptive evolutionary change over two pairs of generations for each of three wild populations. We found significant genetically based increase in mean fitness in five of the six cases, even as environmental differences between years tended to reduce mean fitness, obscuring the genetic increase. This direct demonstration of adaptation makes clear that it is an ongoing process ameliorating the effects of environmental deterioration on mean fitness of populations.

## Introduction

Adaptive evolution is amply documented, yet understanding of the pace of adaptation as it proceeds remains limited. It has become clear that natural selection can demonstrably change populations faster than Darwin (1859) imagined. Numerous examples document recent rapid evolutionary adaptation to new environments in the wild. These include evolution of resistance or tolerance of metals in soils (Antonovics & Bradshaw, 1970; McNeilly, 1968), predators (Reznick et al., 1995), severe drought (Franks et al., 2007) and associated changes in food availability (Grant & Grant, 2003), and herbicides (Kreiner et al., 2019), all within 5–20 generations. These findings imply that the populations harbored capacity for ongoing adaptation to the particular novel, adverse conditions they encountered. Given these and other studies, rapid adaptation to drastic environmental change is no longer surprising, though it is not guaranteed. Under apparently more typical environmental conditions, however, much less is known about whether selection occurs, and, if it does, whether and at what pace adaptation proceeds. Genomic approaches have demonstrated selection-induced changes, both within and between successive generations, in allele frequency throughout genomes, e.g., for *Drosophila melanogaster* (Bergland et al., 2014), and for *Mimulus guttatus* (Monnahan et al., 2021). These cases imply ongoing adaptation, yet the pace of change in mean fitness of populations, an issue that is critical to understanding ongoing adaptation and population persistence in nature, remains unclear.

It is now widely recognized that, for sexually reproducing populations, the additive genetic variance of fitness is the substrate for genetically based adaptation, i.e., change in mean fitness. Fisher (1930), considering the effects on fitness of a single genetic locus, formalized this idea as the fundamental theorem of natural selection (FTNS). The validity and precise interpretation of Fisher’s result have been debated and contested (Bürger, 2000; Ewens, 2004, 2024; Okasha, 2008). Variation in individual fitness within a population is highly polygenic; accordingly, the later developed quantitative genetic analog of FTNS (Frank, 2012; Price, 1970, 1972; Rice, 2004) serves as an approximating model for evolutionary adaptation. For populations growing in their usual habitat, additive genetic variance for fitness was long considered to be typically nil, or nearly so (e.g., Charlesworth, 1987). The challenges of estimating this property are great, and few direct estimates have been published for wild populations, most of them very recently (Etterson, 2004a; Bonnet et al., 2022; Kulbaba et al., 2019; Peschel & Shaw, 2024; Schoen & Speed, 2024; Sheth et al., 2018; Torres-Martinez et al., 2019).

These clear cases demonstrating additive genetic variance for fitness indicate substantial standing genetic variation that could, in principle, support ongoing adaptation, yet hindrances to adaptive evolution are manifest (Antonovics, 1976a; Bradshaw, 1991). Moreover, even as evidence of standing additive genetic variance for fitness builds, the accuracy of quantitative predictions of adaptive rate based on FTNS remains unknown. Besides aspects of mathematical interpretation and applicability (e.g., Ewens, 2024), the theorem ignores both environmental variation and evolutionary processes other than selection. These points highlight persistent questions: (a) To what extent do populations harbor capacity for ongoing adaptation? (b) Do predictions of evolutionary response to selection imply that populations will remain stable or grow? (c) Regardless of the accuracy of predictions of its rate, to what extent does selection-based increase in mean fitness mitigate effects of environmental deterioration on absolute fitness? These questions are central to understanding adaptive evolution at any time; they are particularly urgent in the present era of threats to biodiversity from extremely rapid change in climate and other aspects of environment (Hoffmann & Sgrò, 2011; Parmesan, 2006).

To address these questions requires empirical studies of focal populations that yield not only estimates of the key parameters, mean fitness, and additive genetic variance of fitness, but also direct assessment of the change in mean fitness realized in the next generation. Individual fitness is profoundly sensitive to environmental influences (Antonovics et al., 1987), as is selection (Antonovics et al., 1988). Accordingly, the most informative estimates of these parameters of a population’s fitness distribution are ones based on data for individuals growing in the wild. Further, experimental randomization of genetic lineages over extant environmental variation (e.g., Antonovics et al., 1987) is necessary to ensure that estimates are free of confounding between genetic and environmental influences. Consequently, realistic answers to the above questions entail experiments in nature that amass fitness records spanning the lifetimes of a representative set of pedigreed individuals (Shaw, 2019). For three populations of the annual legume, *Chamaecrista fasciculata*, growing in their source sites, Kulbaba et al. (2019; see also, Geyer et al., 2022) addressed question (a), finding substantial additive genetic variance for fitness. As evidence addressing question (b), the approximate prediction of the next generation’s population mean fitness, quantified as the number of seeds produced per individual seed planted, was near 1 or greater in all cases, yielding the expectation that the populations would remain similar in size or grow.

Here, we address question (c), to what extent does genetically based change in mean fitness due to selection mitigate effects of environmental change on fitness? We leverage the experimental field plantings used to address questions (a) and (b). Into those plantings of pedigreed cohorts, we also planted the progeny from each cohort whose mean fitness and additive genetic variance for fitness are reported in Kulbaba et al. (2019). For each cohort, parental and progeny, we have obtained unusually complete fitness records, from seed planted, through germination and survival, to number of seeds produced, with the goal of directly accounting for selection as thoroughly as feasible.

## Materials and methods

**Figure 1.**
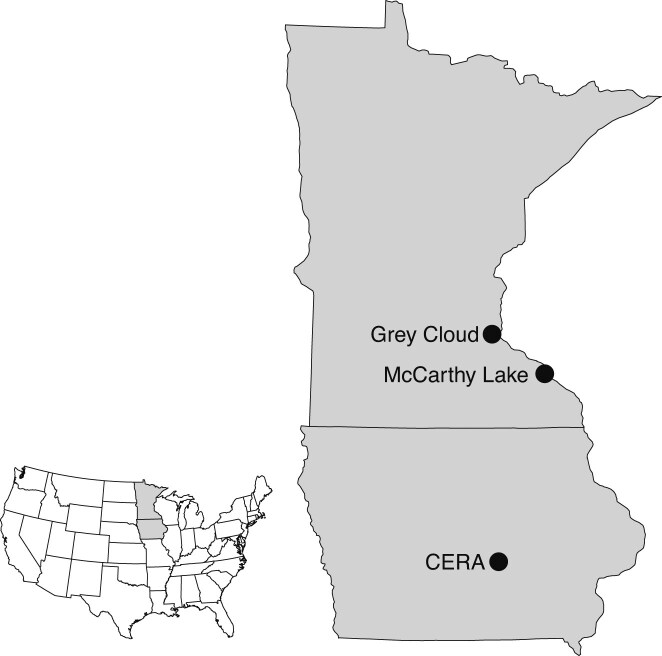
Map of locations where populations of *Chamaecrista fasciculata* were sampled and experimental populations were grown.

We summarize here the methods, which are given in greater detail in Sheth et al. (2018) and Kulbaba et al. (2019). Our study focused on three populations of *C. fasciculata* (Fabaceae, partridge pea), which is native to much of eastern North America (Figure 1). Through this large region, it occupies a wide range of conditions; in Minnesota (MN) and Iowa (IA), where we conducted our study, it grows in grasslands, especially on relatively sandy soils (Stanton-Geddes et al., 2012a, b). The plant is hermaphroditic, apart from rare incidence of male-sterility (Williams & Fenster, 1998). It is bee-pollinated with a variable mixed-mating system (Kulbaba & Shaw, 2021). Formal genetic crosses on a large scale have proven tractable (Etterson, 2004a; Kulbaba et al., 2019). Its annual life-cycle and limited seed dormancy (Fenster, 1991b) suit it well to lifetime accounting of fitness in terms of the number of offspring an individual contributes to its population (e.g., Stanton-Geddes et al., 2012a). It has been a focus of extensive prior evolutionary research, including studies of gene flow (Fenster, 1991a, b; Fenster et al., 2003), outbreeding depression (Erickson & Fenster, 2006; Fenster & Galloway, 2000), local adaptation (Etterson, 2004a; Galloway & Fenster, 1999, 2000), capacity for adaptation to changing climate (Etterson & Shaw, 2001; Etterson, 2004a, b; Peschel & Shaw, 2024; Peschel et al., 2021), and evolution of range limits (Stanton-Geddes et al., 2012a, b).

### Population sampling and genetic crosses

**Figure 2.**
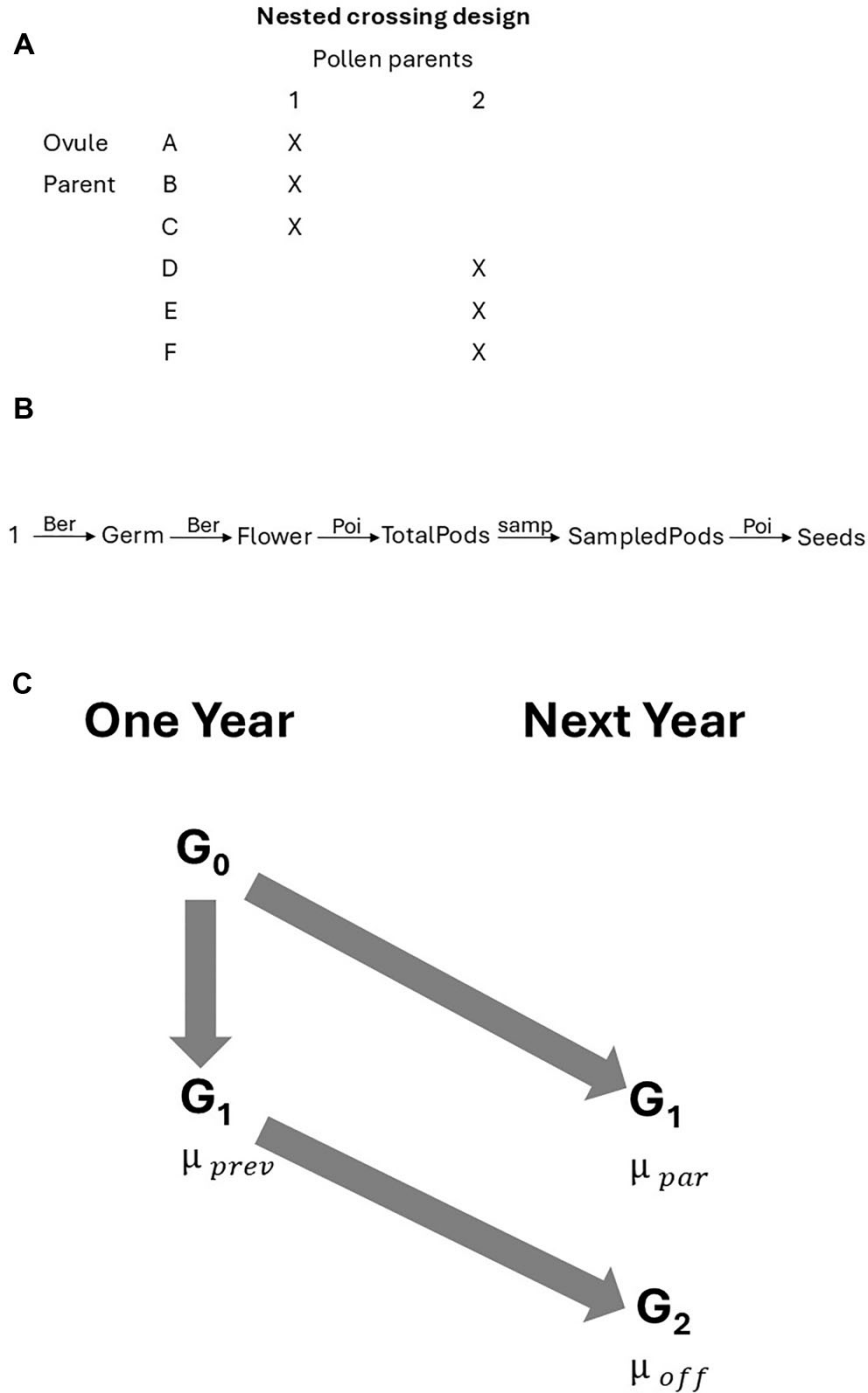
(A) Design of experimental crosses, as outlined in Falconer and Mackay (1997, p.165). Here, only two paternal individuals and six maternal individuals are shown. The actual numbers of individuals in each cohort are given in Table S1b.) (B) Graphical model for fitness of *Chamaecrista fasciculata*, implemented in the aster analyses. The initial node, 1, represents an individual planted as a seed; Germ indicates its germination state (0 or 1); Flower indicates whether it flowered (0 or 1); TotalPods indicates the number of pods it produced; SamplePods is the number of pods for which seeds were counted; Seeds is the number of seeds found in that sample. The probability distribution assigned for each transition is given above each arrow: Ber is Bernoulli; Poi is Poisson. (C) Overview of the scheme of the field experiment, shown for a single population evaluated in two consecutive years (One Year, Next Year). G1 seeds were obtained from plants growing in the greenhouse from seeds collected in the field (G0) and hand-pollinated in the design shown in Figure 2A. A full subset of G1 was planted in the Fall one year, and a replicate full subset of G1 was planted the following Fall. In each case, fitness components of individuals were recorded during the ensuing growing season. In addition, in the Fall of the second year, the offspring (G2) of G1 grown in the previous year were also planted and measured in the following year. For each population, altogether three replicate (annual) cohorts of G1 were grown. The second generation (G2) was grown from the seeds that each of the first two G1 cohorts produced.

Samples of seeds were collected from each of three sites: two in Minnesota at Grey Cloud Dunes and Kellog-Weaver Dunes (Scientific and Natural Areas, held by MN Department of Natural Resources, the latter jointly held by The Nature Conservancy) and one in Iowa at the Conard Environmental Research Area (CERA, held by Grinnell College). At the MN sites, we sampled 200 individuals, and at CERA 100 individuals, at least 10 m apart. From each plant sampled in the field, a single individual was grown (G0) in a greenhouse at the University of Minnesota, with locations assigned at random. From these plants, we generated a pedigreed set of offspring representing each population, in a standard quantitative genetic crossing design (Figure 2A; see also, Falconer & Mackay, 1996, p. 166f.; Lynch & Walsh, 1998, pp. 570–576), as follows. Using pollen from each individual randomly designated to serve as a pollen parent (sire), we hand-pollinated numerous, previously emasculated flowers on each of a distinct set of randomly assigned individuals from the same source population. In this way, we obtained three full-sib groups for each pollen donor. For each of the populations the number of pollen parents was: Grey Cloud: 42; Kellog-Weaver Dunes: 48; and CERA: 21.

### Experimental fitness assessment in source sites

From these crosses, we obtained sufficient seed (G1) for plantings into fields closely resembling and near to the source site of each population (< 500 m away) in each of 3 years. To avoid confusion between our experimental plants and naturally germinating recruits, we did not plant into the precise source locations. However, we chose to plant near to each population’s source location, into existing vegetation and without manipulating environmental conditions, in order to assess ongoing adaptation in conditions as similar as feasible to those typically impinging on each population. At the outset, within each site, numerous 50-m transects were marked such that a subset could be chosen at random each year to be planted. We planted G1 replicates in October–November 2014, 2015, and 2016. At 2 m spacing along the transects, full-sib families (i.e., maternal sibships) were assigned at random to planting locations where, within the existing vegetation, 5 seeds were planted with 10 cm spacing. Each full-sib family was typically represented at six locations randomly distributed throughout, i.e., 30 seeds per full-sib family (see Table S1 for more details on genetic representation in each site and year). We refer to each cohort according to the year the plants grew, i.e., the year after the seeds of that cohort were planted.

Through frequent visits each season following planting, we recorded fitness components for each plant: whether it germinated, whether it survived to flower, the number of flowers it produced, the number of fruits produced, and the number of seeds it produced through open pollination (Figure 2B). For each individual, we enclosed maturing fruits in mesh bags to catch seeds as fruits dehisced. In cases where seeds could not be counted for all fruits, we recorded the number of fruits subsampled to obtain counts of seeds. Our analysis, detailed below, yields fitness estimates as the number of seeds produced through maternal function per seed planted, corresponding to *per capita* seed production. Because the data encompass a full life-cycle assessment of components of fitness, including overwinter survival to germination, the estimates of mean fitness approximate population growth rate for the given cohort.

To evaluate the average fitness of the offspring generation (G2), we planted the seeds into locations assigned at random to maternal sib groups, within a separate randomly chosen set of transects among the G1 plantings in Fall 2015 and 2016. This evaluation of fitness of offspring growing contemporaneously, and randomized together, with a replicate cohort of G1 avoids confounding of genetic change with environmental difference, and thus enables direct comparison of mean fitness estimates to assess the contribution of each to change in mean fitness. This experiment can thus be viewed as a “resurrection” study, though these tend to allow elapse of more generations between cohorts, where the genealogical connection between them is not directly known (Franks et al., 2008; Pennington et al., 2025). It was not feasible to plant all G2 seeds; the proportion of seeds planted from each family was taken into account in estimates of mean fitness (see below). We obtained the full fitness records of individuals in these G2 cohorts (2016, 2017; Figure 2C), blinded with respect to cohort and family identities. In analyses detailed below, we used methods similar to those for addressing subsampling of fruits of G1 plants (noted above).

We note that the G2 cohorts were produced via open-pollination of G1, and sires of individual G2 seeds are not known. Accordingly, it is important to consider the impact on fitness of seed production via paternity, as well as the extent of inbreeding and, thus, possible role of inbreeding depression. In a separate field experiment, Kulbaba & Shaw (2021) inferred seed paternity using molecular markers and estimated outcrossing rate exceeding 70% and biparental inbreeding less than 10% for *C. fasciculata* growing at planting density similar to this study. Thus, despite the potential for selfing and mating between relatives, we expect inbreeding of G2 offspring to be modest. Kulbaba & Shaw (2021) also found no evidence for a genetically based tradeoff between offspring production via maternity and paternity. The relationship between seed production via maternity and paternity was positive, and varied depending on planting density. As a further consideration, in populations with age-structure, fitness depends on timing of offspring production. In an increasing population, earlier produced offspring make greater contribution to fitness (see e.g., Charlesworth, 1980, section 5.3). Here, however, with an annual plant having a single cohort per season, the within-season timing of producing offspring does not bear directly on fitness accounting.

### Statistical analysis

The statistical analyses outlined below and detailed in Geyer et al. (2025) were carried out using R package aster (Geyer, 2025) in R version 4.5.1 (R Core Team, 2025) and are fully reproducible from raw data to estimates presented here using the archived data and R markdown code.

Using the G1 fitness records, including for the many individuals that either did not germinate or did not survive to reproduce, we used aster analysis for mixed models (Geyer et al., 2013) to estimate additive genetic variance for fitness (V_a_(W)), as well as the genetic effects on fitness for each G0 sire family, as reported in Kulbaba et al. (2019). Lacking information about identities of pollen sources for seeds produced by G1 and G2 cohorts, as noted above, and also to avoid confounding of maternal effects with additive genetic effects, our analysis of genetic effects is based on sires in G0, i.e., paternal parents of G1, because we expect the analysis according to paternal lineage, rather than maternal lineage, to minimize biases due to non-genetic transmission.

Similarly, for each G2 cohort, we carried out mixed effects aster analysis of the fitness records of G2 individuals to estimate the genetic effects on fitness for each lineage in the G2 cohort, i.e., descendants of each individual used as a pollen parent in G0. We used these estimated genetic effects in G1 and G2 in the calculations described below.

### Mean fitness and partitioning the change in it

Our estimates of mean fitness and changes in mean fitness involve two concepts: (a) familial mean fitness parameters, where an individual family refers to descendants of a particular sire (pollen parent) in G0, and (b) the Price (1970) equation and closely related ideas, as explained below. Whereas W is typically used to denote fitness in evolutionary theory, our notation uses *μ* to refer to estimates of fitness based on different subsets of data, as defined by the equations below, with subscripts to distinguish particular cases.

Familial mean fitness parameters are obtained as the point estimates of random effects obtained in the mixed effects aster analysis transformed through the mapping from the canonical parameter scale of the EFMM (exponential family mixed model, here, aster models with random effects) to the mean value parameter scale (i.e., the measurement scale, in units of number of seeds produced per individual seed planted). These familial mean fitness parameters roughly correspond to additive genetic effects in classical quantitative genetics (in which, in contrast to the case here, every quantity is assumed jointly multivariate normal, hence an LMM [linear mixed model]). We note that, here, familial effects are effects of a (G0) sire in the analysis of G1 data or (G0) maternal grandsire effects in the analysis of G2 data,

Following Price (1970, 1972) and others (Lande & Arnold, 1983; Rice, 2004, chapter 6; Frank, 2012), we take the change in mean of a trait, $\theta $, due to selection to be given by


(1) \begin{eqnarray*}
\Delta {\mathrm{mean}}\left( \theta \right) = {\mathrm{mean}}\left( {\theta \cdot \frac{\mu }{{{\mathrm{mean}}\left( \mu \right)}}} \right) - {\mathrm{mean}}\left( \theta \right),
\end{eqnarray*}

where $\theta $ refers to any numerical trait and $\mu $ is any measure of fitness. The right-hand side of equation (1) is just the difference between a weighted mean and an unweighted mean. It is valid for any vector $\theta $ and any vector $\mu $ having nonnegative components that are not all zero. It gives the change in means when the items being averaged (trait values) do not change but their representation does change. Equation (1) describes any such situation. Often equation (1) is rewritten as


(2) \begin{eqnarray*}
\Delta {\mathrm{mean}}\left( \theta \right) = \frac{{{\mathrm{cov}}\left( {\theta ,\mu } \right)}}{{{\mathrm{mean}}\left( \mu \right)}}.
\end{eqnarray*}

Equality of the right-hand sides of equations (1) and (2) follows from the definitions of mean and covariance.

For the special case where the trait is fitness itself (so $\theta $ is $\mu $), equation (1) becomes


(3) \begin{eqnarray*}
\Delta {\mathrm{mean}}\left( \mu \right) = {\mathrm{mean}}\left( {\mu \cdot \frac{\mu }{{{\mathrm{mean}}\left( \mu \right)}}} \right) - {\mathrm{mean}}\left( \mu \right).
\end{eqnarray*}

And equation (2) becomes


(4) \begin{eqnarray*}
\Delta {\mathrm{mean}}\left( \mu \right) = \frac{{{\mathrm{var}}\left( \mu \right)}}{{{\mathrm{mean}}\left( \mu \right)}}.
\end{eqnarray*}

There are different possibilities for what can be used for $\mu $ in these equations. Analyses in Lande & Arnold (1983) used individual survival. If $\mu $ were taken to be additive genetic fitness effects, then equation (4) is the conclusion of Fisher’s (1930) FTNS for the single locus case (Rice, 2004, p. 181). We use familial effects, our approximation to additive genetic effects, in which case our use of equation (4) approximates the FTNS for the environment of that particular year. We also have the interpretation of equation (3). This estimates the genetic change in mean fitness due to selection over the duration of a cohort.

A similar equation can be used to obtain the overall change in mean fitness between generations. As noted above, not all offspring produced in the G1 generation were planted for the G2 generation. The representation of the families at planting must be adjusted to their frequencies, as produced, to properly account for the subsampling at the time of planting. This adjustment is similar: the correct frequencies for the families are proportional to their fitnesses. Hence:


(5) \begin{eqnarray*}
\Delta {\mathrm{mean}}{{\left( \mu \right)}_{{\mathrm{total}}}} = {\mathrm{mean}}\left( {{{\mu }_{{\mathrm{off}}}} \cdot \frac{{{{\mu }_{{\mathrm{prev}}}}}}{{{\mathrm{mean}}\left( {{{\mu }_{{\mathrm{prev}}}}} \right)}}} \right) - {\mathrm{mean}}\left( {{{\mu }_{{\mathrm{prev}}}}} \right),
\end{eqnarray*}

where here ${{\mu }_{{\mathrm{off}}}}$ is the familial fitness in a given offspring (G2) subexperiment (site–year combination) and ${{\mu }_{{\mathrm{prev}}}}$ is the familial fitness in the respective parental (G1) subexperiment in the previous year, containing the actual maternal parents of those offspring. This is not a straightforward difference of means because the offspring were not planted in the frequencies that they were produced. The term ${{\mu }_{{\mathrm{prev}}}}/{\mathrm{mean}}( {{{\mu }_{{\mathrm{prev}}}}} )$ corrects for this experimental manipulation of the representation of families.

We decompose the total change in mean fitness, given by equation (5), as the sum of three terms, labeled selection, environmental, and residual. The first of these is equation (3), rewritten with subscripts as


(6) \begin{eqnarray*}
\Delta {\mathrm{mean}}{{\left( \mu \right)}_{{\mathrm{selection}}}} = {\mathrm{mean}}\left( {{{\mu }_{{\mathrm{prev}}}} \cdot \frac{{{{\mu }_{{\mathrm{prev}}}}}}{{{\mathrm{mean}}\left( {{{\mu }_{{\mathrm{prev}}}}} \right)}}} \right) - {\mathrm{mean}}\left( {{{\mu }_{{\mathrm{prev}}}}} \right),
\end{eqnarray*}

which is the change in mean fitness due to genetic selection within a generation, in this case the G1 generation. This is equation (3) with ${{\mu }_{{\mathrm{prev}}}}$ inserted for $\mu $. To obtain the change in mean fitness due to difference in environment between generations, we use, for the first term, the estimate of mean fitness for the pedigreed cohort in the succeeding year. Thus, the change in mean fitness due to difference in environment is


(7) \begin{eqnarray*}
\Delta {\mathrm{mean}}{{\left( \mu \right)}_{{\mathrm{environmental}}}} = {\mathrm{mean}}\left( {{{\mu }_{{\mathrm{par}}}}} \right) - {\mathrm{mean}}\left( {{{\mu }_{{\mathrm{prev}}}}} \right),
\end{eqnarray*}

where ${{\mu }_{{\mathrm{par}}}}$ and ${{\mu }_{{\mathrm{prev}}}}$ are the familial fitnesses in the pedigreed (G1) individuals grown in year 2 and year 1, respectively. The change in mean fitness due to selection and to environment need not sum to the overall change in mean fitness between generations. The residual component is the remainder


(8) \begin{eqnarray*}
\Delta {\mathrm{mean}}{{\left( \mu \right)}_{{\mathrm{residual}}}} &=& \Delta {\mathrm{mean}}{{\left( \mu \right)}_{{\mathrm{total}}}} - \Delta {\mathrm{mean}}{{\left( \mu \right)}_{{\mathrm{selection}}}}\\&& \,- \Delta {\mathrm{mean}}{{\left( \mu \right)}_{{\mathrm{environmental}}}}
\end{eqnarray*}

We present the estimates of mean fitness for each cohort of G1, and the estimates of mean fitness for the corresponding G2 cohort. Standard errors of these estimates were obtained using approximate Fisher information for parameters derived in section 2 of Geyer et al. (2013) and the delta method, as illustrated in Geyer et al. (2026). We note that estimates of standard errors for change due to selection, because they involve data from only one cohort, are much smaller than the other standard errors, which involve data from two cohorts (analyzed as if the data were independent). We display the changes in mean fitness, as partitioned into the three components described above. The analysis in Geyer et al. (2025) corrects two cases of undefined standard errors of Va(W) in Geyer et al. (2022), and recovers all other values as given there.

## Results

### Mean fitness of pedigreed cohorts (G1)

**Figure 3.**
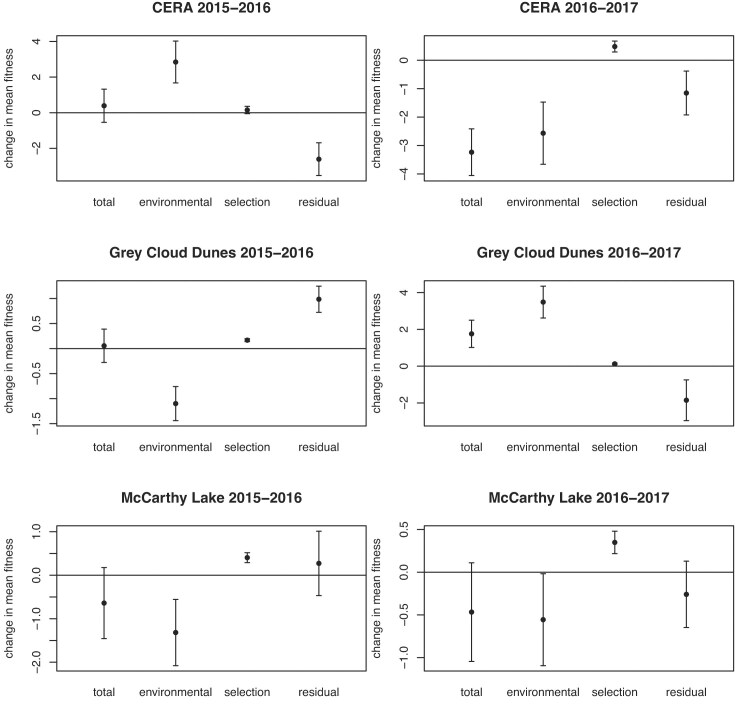
Estimates (with 95% confidence intervals) of the components of the change in mean fitness from parent (G1) to offspring (G2), in units of number of seeds produced per seed planted, for three populations over two generational cycles. “Total”: the difference of the mean fitness of the offspring cohort (G2) from that of its parents (G1), “Environment”: the difference in successive years between mean fitness of replicate cohorts of the parental (G1) generation, “Selection”: the change in mean fitness within the parental (G1) generation, and “Residual”: the amount of the Total change in mean fitness between generations that remains after accounting for Selection and Environment.

By growing genetic replicates of progeny of experimental crosses (G1) in the source site of each population in each of 3 years, we assessed the average fitness of each population as expressed in each year, as the average number of seeds it produced per seed planted, taking into account germination, survival, flowering, and seed production. In most cases (six out of nine), the estimate of mean fitness in the year the cohort was grown is greater than 1 (Table 1), indicating that the populations would maintain their size or increase numerically into the next generation. In three cases (a single year for each population), $\mu $, was estimated as less than 1 (0.7–0.92; see Table 1), though, given the uncertainty of the estimates (i.e., standard error), we cannot rule out 1. These estimates suggest that the population subject to the environmental conditions in that year may decline in size, in the absence of adaptive evolution. For each population, $\mu $ differed significantly among the genetic replicates grown in different years (Table 1), indicating interannual environmental variation that substantially affected overall fitness. The differences in $\mu $ between genetic replicates grown in successive years, indicating changes in average effects of environment, i.e., Δ$\mu $_environmental_, were not consistent in direction (Figure 3).

**Table 1.** Estimates of G1 mean fitnesses within the season each cohort is grown. Mean fitness is in units of number of seeds produced per seed planted. Estimates that differ from 1 by more than 1.95 times their standard error are considered significantly different from 1 (corresponding to population replacement) at level 0.05. The “before selection” column is the ${\mathrm{mean\ }}( \mu )$ term in equation (3). The “after selection” column is the ${\mathrm{mean\ }}( {\mu \cdot \mu /{\mathrm{mean}}( \mu )} )$ term in that equation. Thus, the “before selection” column is the average of the estimated fitnesses of the families, and the “after selection” column is the weighted average of the same, with weighting proportional to the fitness of each family. Year refers to the year the plants grew, which is the year immediately after the cohort was planted as seeds.

	Mean fitness of parental populations				
		Before selection		After selection	
Site	Year	Estimate	Std. error	Estimate	Std. error
CERA					
	2015	0.795	0.433	0.947	0.537
	2016	3.641	0.414	4.123	0.500
	2017	1.076	0.374	1.186	0.442
Grey Cloud Dunes					
	2015	2.022	0.143	2.190	0.154
	2016	0.923	0.098	1.048	0.119
	2017	4.409	0.431	4.902	0.513
McCarthy Lake					
	2015	2.720	0.296	3.124	0.283
	2016	1.403	0.252	1.751	0.316
	2017	0.846	0.110	1.078	0.134

### Mean fitness of offspring cohorts (G2)

Most offspring cohorts expressed mean fitness of nearly 1 or substantially higher, yielding the expectation that the populations would maintain their size or increase into the next generation. There was a single exception: the 2017 G2 cohort of CERA had a mean fitness of 0.4, indicating population decline from 2017 into 2018 to less than half its number.

### Intragenerational response to selection

Every pedigreed replicate (G1) of each population expressed strong evidence for Va(W) (Kulbaba et al., 2019). Thus, each population harbored capacity for ongoing adaptation to the prevailing environmental conditions, and was, in fact, undergoing genetically based natural selection. Correspondingly, in each G1 cohort except one (CERA, 2015–2016), the intragenerational genetic change in mean fitness was significantly greater than zero, directly demonstrating genetic adaptation through the duration of the cohort (Figure 3, Table S2). However, they are generally lower, considerably so, than the FTNS predictions (Geyer et al., 2022, also presented in Table S2, with estimates from 2022 unchanged with the exception of two standard errors, previously reported as infinite, now corrected). Thus, adaptation was realized within each pedigreed (G1) cohort, but it fell short of the approximation from the FTNS.

### Intergenerational change in mean fitness

For each pair of G1 and G2 cohorts, the mean fitness of the progeny cohorts (Table 2) reflects the often substantial environmental differences between successive growing years, as seen in the above comparison of $\mu $ between successive pairs of G1 cohorts, i.e., Δ$\mu $_environmental_, in addition to the genetically based change within the G1 generation (Figure 3). Partitioning the overall change in $\mu $ from G1 to G2 (as described in the section “Methods”), we find that the change due to the difference in environment (averaged over all the families) generally exceeds, often dramatically, that due to the genetic change within the G1 cohort, sometimes in the same, but often in opposite, directions. In general, the sum of these two components does not closely match the actual difference in mean fitness between parents and their progeny. The discrepancy in most cases exceeds, in absolute magnitude, the intragenerational genetic change in mean fitness, as shown in Figure 3.

**Table 2.** Estimates of mean fitnesses of G2, the offspring cohorts. Mean fitness is in units of number of seeds produced per seed planted. Estimates that differ from 1 by more than 1.95 times their standard error are considered significantly different from 1 (corresponding to population replacement) at level 0.05. Year refers to the year the plants grew, which is the year after the cohort was planted as seeds.

	Mean fitness of offspring populations		
Site	Year	Estimate	Std. error
CERA			
	2016	1.186	0.197
	2017	0.406	0.075
Grey Cloud Dunes			
	2016	2.077	0.090
	2017	2.682	0.366
McCarthy Lake			
	2016	2.080	0.279
	2017	0.935	0.151

## Discussion

Here, we have investigated evolutionary adaptation for each of three populations of *C. fasciculata* expressed in distinct environmental conditions that naturally occurred in each of 3 years. To do so, we experimentally disentangled genetic and environmental contributions to change in mean fitness. Consistent with the significant V_a_(W) we previously found (Geyer et al., 2022; Kulbaba et al., 2019), adaptive capacity was realized in significant genetically based increase in mean fitness within each G1 cohort, with a single exception (CERA, 2015–2016). From one year to the next, however, environmental change depressed mean fitness, in some cases. Nevertheless, adaptive response was pervasive in the progeny cohorts, though this response showed complexities. We have found that V_a_(W), though it is a theoretically sound metric of adaptive capacity (Rice, 2004), yielded only a rough approximation of the genetically based change in change in mean fitness under the environmental differences that played out between generations. Crucially, even though environmental differences between generations can obscure genetic adaptation, we found instances in which the realized genetically based adaptation ameliorated the effect of environmental deterioration. Moreover, we have found an additional substantial, but still less predictable contributor (Residual) to change in mean fitness between generations.

The prospect and process of adaptation tends not to be considered when, as here, environmental conditions seem benign and relatively stable. Despite apparent stability of the environment, mean fitness of the replicate G1 cohorts differed substantially among years. In some years, mean G1 fitness fell below replacement, though not significantly so. Thus, none of the cases documented here are clear instances of evolutionary rescue, i.e., restoration of positive growth rates through genetic adaptation (Gomulkiewicz & Holt, 1995). Nevertheless, our findings directly demonstrate adaptation as a generationally ongoing process, even when it may not be apparent.

Retrospective studies have now yielded numerous examples of past adaptation over few generations (e.g., 5–20), especially in cases of drastic change in environment (Franks et al., 2007; Kreiner et al., 2019; McNeilly, 1968), but prospective studies of ongoing and future adaptation have been few. Change in allelic representation within generations and between successive ones has been shown (Bergland et al., 2014; Monnahan et al., 2021), but direct evidence demonstrating the pace and process of ongoing genetically based adaptation, in the sense of change in mean fitness, along with its sensitivity to interannual variation, has been limited. The long-standing view that V_a_(W) is generally nil has underlain doubts about whether biologically meaningful adaptation proceeds generationally, leading to the distinction between “ecological time” and “evolutionary time.” This study (along with Peschel & Shaw, 2024 and Nashoba, 2016) shows that these are not distinct timeframes, a point that Antonovics (1976b) emphasized. Here, evolutionary adaptation proceeded over the life cycle of each G1 cohort subject to the environmental conditions prevailing over that particular year in the respective site.

The FTNS has served as a valuable conceptualization for considering rates of ongoing genetic adaptation. The theory, and its interpretation, has been explicated by numerous scholars (e.g., Edwards, 1994, 2014; Frank, 1997; Okasha, 2008). Full agreement about interpretation has not emerged, and Ewens (2024) has recently raised concerns about the mathematical correctness of the single-locus theory. Nevertheless, the FTNS remains useful in its quantitative genetic formulation (Frank, 2012; Price, 1970, 1972; Rice, 2004). Understandably, the derivation implicitly concerns a uniform, constant environment; it does not account for vagaries of environmental variation and change. We emphasize, moreover, that additive genetic variance for fitness is a theoretical quantity that, partly because of its derivation based on Gaussian effects, does not correspond precisely to its estimate from the statistical analysis accommodating inherently non-Gaussian fitnesses (as also noted in Kulbaba et al., 2019 and Geyer et al., 2022).

Empirical efforts to apply FTNS have been very few (Bonnet et al., 2022; Kulbaba et al., 2019; Nashoba, 2016; Peschel & Shaw, 2024) and comparisons of realized to predicted adaptation fewer still (Nashoba, 2016; Peschel & Shaw, 2024). The six cases presented here greatly expand the available comparisons of realized adaptation to the FTNS prediction. As qualitatively predicted by FTNS, intragenerational genetic adaptation was realized in each case, but its magnitude fell short of the predicted increase (Table S2). In part, this discrepancy could arise because frequency distributions of fitness do not conform to Gaussian distributions typically suitable for quantitative traits. Fitness distributions are typically multimodal and skewed, such that neat partitioning of phenotypic variance into underlying components, as for traits based on Gaussian distributions, does not hold. Joint analysis of components of fitness via aster modeling employs exponential family models with random effects, rather than the linear models appropriate for Gaussian traits. Besides this, the mean fitness of the offspring generation deviated from the FTNS prediction due to interannual difference in environment, which is not easily discernible but is captured through our experimental approach.

Besides the average effect of environment on mean fitness, an additional important contributor to the discrepancy between predicted and realized mean fitness of G2 is substantial interaction between genotype and annual environment affecting fitness expression, which we have previously documented (Figure 3 of Geyer et al., 2022; Kulbaba et al., 2019, reprinted in Figure S1). Relative to one another, families differed in their average fitness between years. Numerous families averaging high fitness in one year expressed relatively poor fitness in the next, and there are also cases of the reverse. Altogether, in half our cases, intergenerational environmental difference resulted in overall decline in mean fitness of progeny, despite the adaptive genetic response within the parental generation. These findings bear on the predictability of fitness in practical contexts, for example, in sourcing of genetic material for restoring populations (Beck et al., 2025). Similarly, in field plantings in two successive years of *Arabidopsis thaliana*, a highly selfing annual plant, Villoutreix et al. (2025) found very low accuracy of predictions of genetic effects on fitness in one year, based on the effects estimated in the other.

The direct demonstration of adaptation presented here makes clear that it should be acknowledged as an ongoing process that, though not apparent, may be maintaining and increasing a population’s mean fitness. There can be no guarantee that adaptation will suffice to ensure evolutionary rescue of populations subject to environmental change (reviewed in Hendry et al., 2018), but inherent capacities for ongoing adaptation should not be dismissed. Here, we assessed adaptation through single, full-generational episodes of selection, using genetic replicates of each population in each of 3 years. It would be additionally informative to evaluate adaptive responses through multiple generations of selection in successive seasons in nature. Such studies could follow adaptive responses that proceed as the genetic composition of the population changes over generations, as in usual experimental studies of artificial selection.

## Supplementary Material

qrag013_Supplemental_File

## Data Availability

Data and code are archived at DOI 10.5281/zenodo.17398467
