# Supplementary material for: Realization of ongoing evolutionary adaptation in the field
Source: Evol Lett. 2026 Apr 11;10(3):315–25. doi: 10.1093/evlett/qrag013 (PMC13229654; doi:10.1093/evlett/qrag013)
Supplement: qrag013_Supplemental_File [file qrag013_supplemental_file.docx]

**Supplementary Material**

Table S1: Representation of genetic families of each population in each year. a) G1, b) G2.

a)

| site | Year | Planted | Sires | Dams |
| --- | --- | --- | --- | --- |
| CERA |  |  |  |  |
|  | 2015 | 1,748 | 20 | 59 |
|  | 2016 | 1,748 | 20 | 59 |
|  | 2017 | 1,750 | 20 | 59 |
| Grey Cloud Dunes |  |  |  |  |
|  | 2015 | 3,658 | 42 | 123 |
|  | 2016 | 3,660 | 42 | 123 |
|  | 2017 | 3,660 | 42 | 123 |
| McCarthy Lake |  |  |  |  |
|  | 2015 | 3,445 | 48 | 132 |
|  | 2016 | 3,447 | 48 | 132 |
|  | 2017 | 3,516 | 48 | 132 |

b)

| site | Year | Planted | Families |
| --- | --- | --- | --- |
| CERA |  |  |  |
|  | 2016 | 1,749 | 18 |
|  | 2017 | 1,753 | 20 |
| Grey Cloud Dunes |  |  |  |
|  | 2016 | 3,651 | 39 |
|  | 2017 | 819 | 32 |
| McCarthy Lake |  |  |  |
|  | 2016 | 3,447 | 38 |
|  | 2017 | 4,066 | 44 |

Table S2. Estimates of the components of the change in mean fitness for each population for each two-generation cycle. Estimates greater than their standard error by a factor of 1.95 are considered significantly greater than zero at level 0.05. Changes in mean fitness are in units of number of seeds produced per seed planted. As described in the text, total is the overall difference between the mean fitness of G1 cohort and its respective offspring, grown in the following year; environmental is obtained as the difference between the mean fitnesses (before selection) of G1 cohorts grown in two successive years; selection is the intragenerational genetic change in mean fitness, i.e. the difference between the mean fitness of the G1 cohort before and after selection over its lifespan; ftns is the prediction obtained from the Fundamental Theorem of Natural Selection (Price 1972), drawn from analyses given in Geyer et al. (2022); residual is the change in mean fitness between parent and offspring that is not accounted for by the sum of the environmental and genetic changes.

| Subset | Change | Estimate | Std. Error |
| --- | --- | --- | --- |
| CERA 2015-2016 |  |  |  |
|  | total | 0.391 | 0.474 |
|  | environmental | 2.846 | 0.599 |
|  | selection | 0.153 | 0.104 |
|  | fftns | 2.697 | 1.210 |
|  | residual | -2.607 | 0.469 |
| CERA 2016-2017 |  |  |  |
|  | total | -3.234 | 0.419 |
|  | environmental | -2.565 | 0.557 |
|  | selection | 0.482 | 0.098 |
|  | fftns | 0.985 | 0.761 |
|  | residual | -1.152 | 0.393 |
| Grey Cloud Dunes 2015-2016 |  |  |  |
|  | total | 0.056 | 0.170 |
|  | environmental | -1.099 | 0.173 |
|  | selection | 0.168 | 0.015 |
|  | fftns | 0.883 | 0.325 |
|  | residual | 0.986 | 0.133 |
| Grey Cloud Dunes 2016-2017 |  |  |  |
|  | total | 1.759 | 0.378 |
|  | environmental | 3.486 | 0.442 |
|  | selection | 0.125 | 0.024 |
|  | fftns | 3.900 | 4.596 |
|  | residual | -1.852 | 0.566 |
| McCarthy Lake 2015-2016 |  |  |  |
|  | total | -0.640 | 0.417 |
|  | environmental | -1.317 | 0.388 |
|  | selection | 0.404 | 0.058 |
|  | fftns | 3.347 | 4.743 |
|  | residual | 0.273 | 0.378 |
| McCarthy Lake 2016-2017 |  |  |  |
|  | total | -0.467 | 0.295 |
|  | environmental | -0.556 | 0.275 |
|  | selection | 0.349 | 0.067 |
|  | fftns | 2.060 | 2.035 |
|  | residual | -0.260 | 0.198 |

Figure S1. [Figure 3 from Kulbaba et al. (2019), as corrected in Geyer et al. (2022)]. Relationship between estimated genetic effects on lifetime fitness (seeds set), as expressed in pairs of years for three

populations of *Chamaecrista fasciculata*. Please note differences in scale across panels.

**
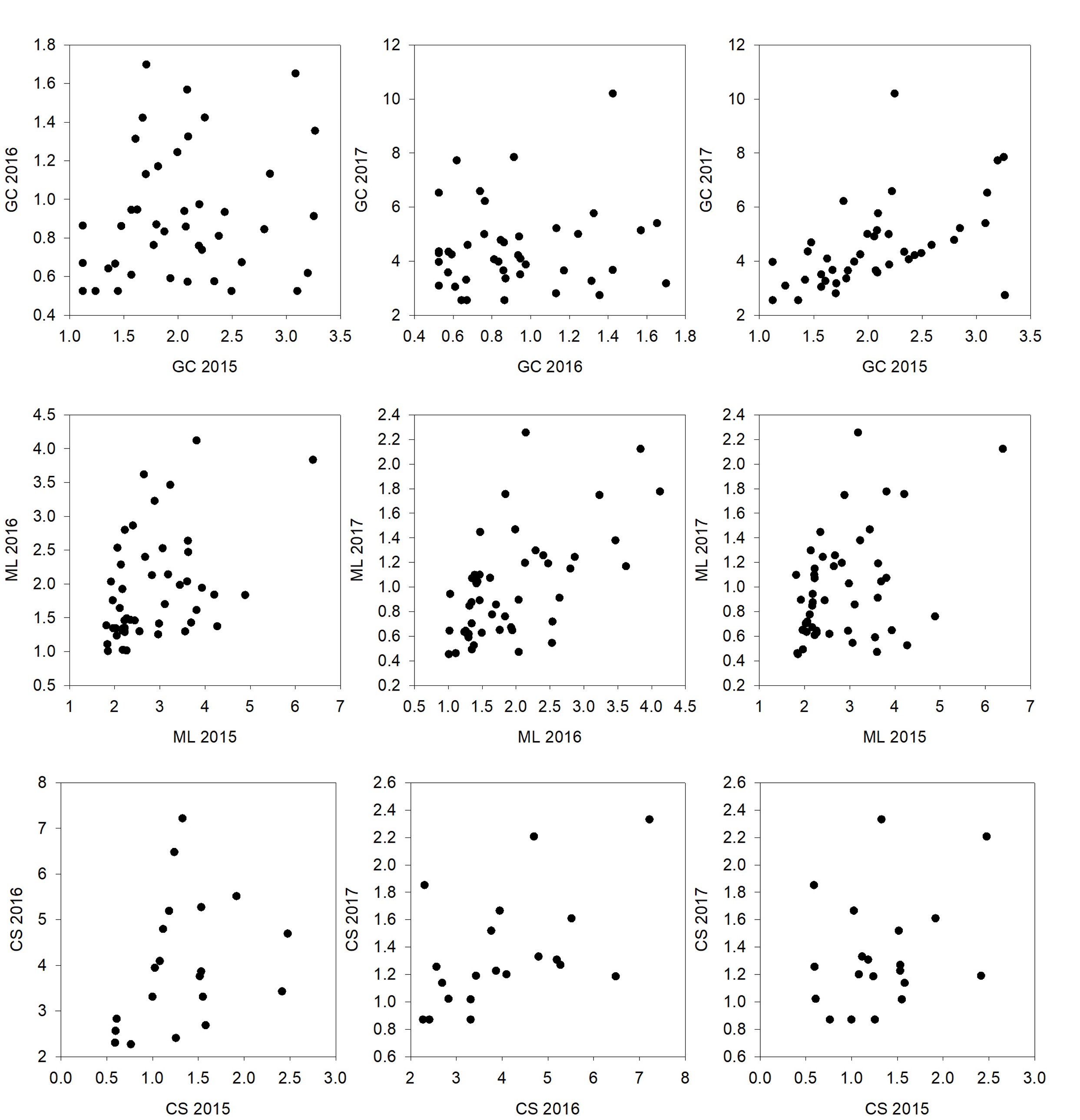
**
